# Commentary on: People search for meaning when they approach a new decade in chronological age

**DOI:** 10.3389/fpsyg.2015.00792

**Published:** 2015-06-09

**Authors:** Erik G. Larsen

**Affiliations:** Department of Political Science, University of Southern DenmarkOdense, Denmark

**Keywords:** meaning in life, aging, age effects, replication, post-publication review, cross-validation

## 1. Introduction

People differ in the extent to which they engage in the search for meaning in life (Steger et al., 2008). Previous research has documented that personal experiences can influence the sense of meaning in life (Machell et al., 2015) and that people report higher levels of searching for meaning in their earlier life stages (Steger et al., 2009). In a recent published article in the *Proceedings of the National Academy of Sciences*, Alter and Hershfield (2014) (abbreviated AH) argue that people are more likely to engage in meaning searching activities as they approach a new decade in chronological age. More specifically, the authors conclude on the basis of six studies, that “adults undertake a search for existential meaning when they approach a new decade in age (e.g., at ages 29, 39, 49, etc.)” (p. 17066). The authors note that the paper “demonstrates a striking pattern in human behavior.” In this commentary I use the empirical material from AH as well as additional data and argue that the patterns demonstrated by AH are open to alternative explanations and lack conclusive evidence^1^.

## 2. Cross-validation of study 1

In Study 1, AH examine whether people having an age ending at nine (29, 39, etc.) is more likely, on average, to question the meaning of life on a four point scale. They conclude, using survey data from the World Values Survey (WVS), that “nine-enders reported questioning the meaning or purpose of life more than respondents whose ages ended in any other digit” (p. 17067). To cross-validate this finding I applied data from all six waves of the WVS with similar measures (compared to one wave used by the authors) and estimated the nine-ender effect as a simple difference in means (as AH), in an ordered logistic regression (to take the ordinal nature of the dependent variable into account) and in a mixed effects model (to treat age as a random covariate).

**Figure 1:**
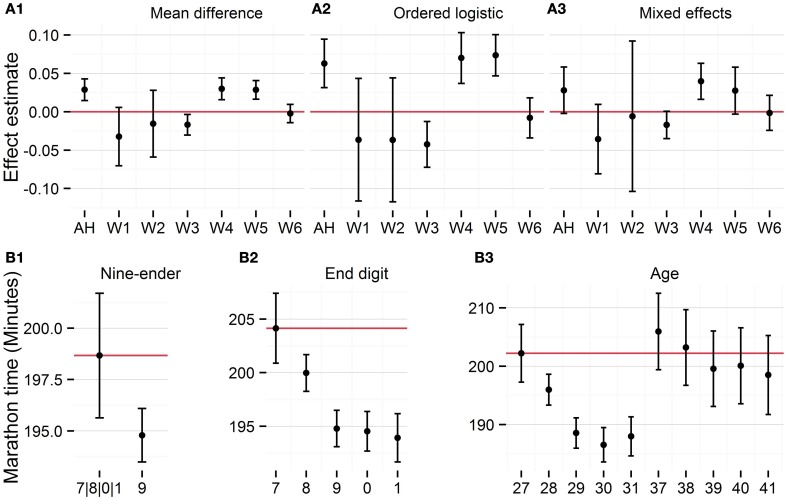
**Validation of nine-ender effect in Study 1 (A1–A3) and Study 5 (B1–B3)**.

In the data set available from WVS, which is updated after the authors wrote their manuscript, there are 61,148 respondents in the age range 25–64. It is not possible to get an identical sample as AH with the data available from WVS. Hence, I report the results using the original data from AH and the six waves available from WVS as of March 26th, 2015. Figures 1A1-A3 show the unstandardized effects of the nine-ender variable in the different models. Overall, contrary to the interpretation made by AH, there is no systematic evidence that nine-enders question the meaning of life significantly more (or less) often.

## 3. Who are the nine-enders? content validity in study 3

In Study 3, the authors argue that nine-enders are more likely to seek extramarital affairs online. Here the authors “categorized 8,077,820 male users, aged between 25 and 64 years, according to the final digit of their ages. There were 952,176 nine-enders registered on the site” (p. 17067) and found that nine-enders were overrepresented at the site. In this study, AH draw inferences of the key independent variable, i.e., nine-ending digits, but this raises concerns related to the validity of the measure. More specifically, examining differences in the age distribution do not enable us to draw strict inferences on the age distribution users had when they signed up at the extramarital affairs dating site, i.e., when they decided to engage in the meaning searching behavior. One should note that this criticism is unrelated to whether or not people self-report their real age when seeking extramarital affairs online, but in other words that a male aged 29 may have signed up at the dating site when he was younger, e.g., 27 years old. Hence, on the basis of the evidence presented by AH, the conclusion that people are more likely to start seeking extramarital affairs when they are nine-enders has notable limitations.

## 4. The robustness of the findings in study 4 and 5

In Study 4, the authors study suicide victims across the United States between 2000 and 2011 and examine whether nine-enders are more likely to commit suicide. The authors estimate an ANCOVA model accounting for specific covariates (region, age, and total deaths) and find empirical support for the expectation [*F*_(1, 1915)_ = 4.35, *P* = 0.037]. However, an ANOVA model (i.e., the same model as AH but without the covariates), show that the relation between suicide rate and nine-enders is non-significant [*F*_(1, 1918)_ = 1.83, *P* = 0.176]. AH provide no motivation for the inclusion of the covariates or examine how the results are conditional upon accounting for the specific covariates^2^. In short, the effect reported by AH is sensitive to alternative model specifications, and one should be cautious when drawing conclusions about the impact of being a nine-ender on suicidal behavior on the basis of the evidence presented by AH.

In Study 5, the authors show that runners who completed marathons ran faster at age 29 and 39 compared to the average of the fastest marathon times from the years before and after. However, these findings are in line with an interpretation of slower marathon times for certain ages. Figure 1B decomposes the nine-ender effect in three random effects models. Figure 1B1 show the nine-ender effect with the other ages as the baseline, i.e., the intercept, and Figures 1B2,B3 estimate the age effect for end digits and all ages, respectively. The results illustrate that the differences in marathon times are unlikely to be affected by a change in running times at 29 and 39, but rather slower running times for people in their late twenties. Hence, when calculating the average of the non-nine-ender times and comparing it to nine-enders, this provide a significant difference but no systematic evidence that nine-enders are faster when they run marathons. In sum, while theoretically intriguing, some of the results presented by AH lack conclusive evidence for a nine-ender effect on the engagement in meaning searching activities.

### Conflict of interest statement

The author declares that the research was conducted in the absence of any commercial or financial relationships that could be construed as a potential conflict of interest.

## Footnotes

^1^Material required to replicate the results presented here is available at http://github.com/erikgahner/fp-meaning.

^2^Using Bayesian model averaging, the posterior probability that the nine-ender variable is different from zero, using the covariates applied by AH, is 27.8%.

## References

[B1] Alter A. Hershfield H. (2014). People search for meaning when they approach a new decade in chronological age. Proc. Natl. Acad. Sci. U.S.A. 111, 17066–17070. 10.1073/pnas.1415086111 25404347 PMC4260584

[B2] Machell K. Kashdan T. Short J. Nezlek J. (2015). Relationships between meaning in life, social and achievement events, and positive and negative affect in daily life. J. Pers. 83, 287–298 10.1111/jopy.12103 24749860

[B3] Steger M. Kashdan T. Sullivan B. Lorentz D. (2008). Understanding the search for meaning in life: personality, cognitive style, and the dynamic between seeking and experiencing meaning. J. Pers. 76, 199–228. 10.1111/j.1467-6494.2007.00484.x 18331281

[B4] Steger M. Oishi S. Kashdan T. (2009). Meaning in life across the life span: levels and correlates of meaning in life from emerging adulthood to older adulthood. J. Posit. Psychol. 4, 43–52. 10.1080/17439760802303127

